# Are Dietary and Serum Advanced Glycation End Products (AGEs) Potential Contributors to Inflammation in Women with Polycystic Ovary Syndrome?

**DOI:** 10.3390/jcm14165803

**Published:** 2025-08-16

**Authors:** Merve Yurt, Hülya Gökmen-Özel

**Affiliations:** 1 Department of Nutrition and Dietetics, Faculty of Health Sciences, Eastern Mediterranean University, North Cyprus Via Mersin 10, 99628 Famagusta, Türkiye; 2 Department of Nutrition and Dietetics, Faculty of Health Sciences, Hacettepe University, 06100 Ankara, Türkiye; hgokmen@hacettepe.edu.tr

**Keywords:** polycystic ovary syndrome, advanced glycation end products (AGEs), dietary AGEs, biochemical parameters, carboxymethyl lysine, soluble receptor for advanced glycation end products, methylglyoxal

## Abstract

**Background/Objectives**: Polycystic ovary syndrome (PCOS) is an endocrine disorder characterized by metabolic and hormonal imbalances in women of reproductive age. Various studies have emphasized that a diet high in advanced glycation end products (AGEs) and high serum AGE levels may be associated with reproductive and metabolic dysfunction in PCOS. Recently, the role played by dietary and serum AGE levels in the pathogenesis of PCOS was emphasized. **Methods**: In this study, we investigated the relationships between dietary AGE intake and serum AGE levels, some metabolic parameters, and anthropometric measurements in individuals with PCOS and a control group of women without PCOS. A total of 87 women with PCOS (*n* = 43) and without PCOS (*n* = 44) of a similar age and with a similar body mass index were included in this study. We analyzed dietary AGE intake, serum AGE (CML, sRAGE, and MGO) levels, and markers of inflammation (TNF-α and hs-CRP). **Results**: The daily dietary AGE intake in the PCOS group (13,191.05 ± 3360.12 kU/day) was higher than that in the control group (11,740.28 ± 2940.61 kU/day) (*p* = 0.035). The serum CML/sRAGE ratio was found to be higher in the PCOS group (413.94 ± 1114.79) than in the control group (143.24 ± 124.71) (*p* = 0.002). The cut-off points for dietary AGE intake, serum CML, and the CML/sRAGE ratio levels, which may be associated with the risk of PCOS development, were determined to be 11,359.06 kU/day, 417.50 ng/mL, and 140.91 ng/mL, respectively. **Conclusions**: Regular monitoring of serum AGE levels may reduce the health risks associated with PCOS. Moreover, to reduce dietary AGE intake in patients with PCOS, we recommend using steaming, boiling, poaching, or simmering with minimal water instead of dry-heat cooking methods.

## 1. Introduction

Polycystic ovary syndrome (PCOS) is an endocrine disorder characterized by irregular menstrual cycles, hyperandrogenism, and polycystic ovary morphology [1]. Polycystic ovary syndrome (PCOS) may adversely affect reproductive health by causing anovulation, infertility, and hyperandrogenism. PCOS may also lead to diabetes mellitus and cardiovascular diseases, as it can increase metabolic complications such as hyperinsulinemia, insulin resistance, and impaired glucose tolerance [2]. The prevalence of PCOS has been reported to range between 6.8% and 12.5%, with a significant increase observed in almost every country over the 30-year period from 1990 to 2019 [1,3]. This increase in the prevalence of PCOS may lead to metabolic disorders and psychological problems that reduce women’s quality of life [3].

Obesity, a sedentary lifestyle, genetic and epigenetic factors, environmental toxins, physical/emotional stress, and unhealthy dietary patterns can influence the development of PCOS [4,5]. The most important nutritional factor is the excessive dietary intake of advanced glycation end products (AGEs). AGEs are heterogeneous irreversible compounds formed by the non-enzymatic glycation of proteins, lipids, and nucleic acids with reducing sugars [6]. Cooking high sugar-, lipid-, and protein-containing foods stimulates non-enzymatic browning reactions and the production of AGEs [7]. The AGE content of animal-derived foods that are cooked at a high temperature or in dry heat for a long time is particularly high [8]. Various studies have emphasized that a high intake of AGEs in the diet and high levels of AGEs in the blood may be associated with reproductive and metabolic dysfunction in women with PCOS [4,5,9]. AGEs cause an increase in inflammatory cytokines such as tumor necrosis factor-alpha (TNF-α), interleukin-6 (IL-6), and interleukin-1β (IL-1β) and inflammation markers such as C-reactive protein (CRP) through the RAGE receptors [2]. The resulting elevation of AGEs in the body may lead to increased oxidative stress in ovarian cells, affecting the levels of steroid hormones secreted by the ovaries. This is closely associated with the development of infertility in women with PCOS [6,10,11,12]. In addition, AGEs in metabolism may inhibit insulin clearance or increase insulin secretion, leading to the development of mechanisms that contribute to insulin resistance [13].

We conducted this study to investigate the relationship between dietary AGE intake, inflammatory parameters, serum AGE levels, and anthropometric measurements in women with and without PCOS.

## 2. Materials and Methods

### 2.1. Study Subjects

In this study, we investigated the relationships between dietary AGE intake, serum AGE levels, metabolic parameters, and anthropometric measurements in individuals with PCOS and a control group comprising women without PCOS.

This case–control study included 43 women (aged 19–31 years) who were diagnosed with PCOS by a gynecologist according to the 2003 Rotterdam criteria and referred to the Eastern Mediterranean University Healthy Living Centre. In this study, we also included 44 women without PCOS (aged 19–29 years) who were matched to the PCOS group in terms of their age and BMI, had no clinical complaints or findings suggesting that they may have PCOS, and had a normal menstrual cycle (18–32 days).

The exclusion criteria included active smoking, pregnancy or breastfeeding, significant weight loss in the last three months, a history of eating disorders, a chronic disease diagnosis, the use of hormonal therapy in the last three months (e.g., hormonal contraception, hormone replacement therapy, or progestogens), and the use of drugs or vitamin supplements that affect glucose metabolism or the lipid profile.

Using G*Power 3.1.9 software with 90% power and a 5% margin of error, we calculated the minimum sample size required for this study to be 82 people in total, with a minimum of 41 participants in each group (case and control). However, given that a 25% loss rate may be experienced during this study, we started with a total of 88 participants (case = 44; control = 44). One participant from the case group was excluded from this study due to missing data. The research procedures were approved by the Clinical Research Ethics Committee of Eastern Mediterranean University (research ethics project number ETK00-2023-0151), and written informed consent was obtained from all participants. During the preparation of this manuscript, the author(s) used ChatGPT (OpenAI, 2024 version) for the purpose of English–Turkish and Turkish–English translation.

### 2.2. Anthropometric Measurements

We measured the body weight and body composition of the individuals using the TANITA MC780 device. Their height was measured using a stadiometer, and their waist and hip circumferences were measured using a non-stretching tape measure [14]. Their body mass index (BMI) was calculated as weight in kilograms divided by height in square meters.

### 2.3. Dietary Assessment

The eating habits of the study participants were evaluated using a food frequency questionnaire (200 items) covering the previous month. The Food and Nutrition Photo Catalog was used to accurately determine the amount of food consumed [15]. The average daily energy and nutrient intakes of the individuals were calculated using the BeBIS v9 program [16].

### 2.4. Dietary AGE Intake

Dietary data were collected using a food frequency questionnaire (FFQ) that assessed the type and amount (g or mL) of each food or beverage consumed in a typical portion over the previous month, as well as the cooking methods (e.g., boiling, grilling, frying, baking) usually used for their preparation. The frequency of consumption was recorded as every meal, every day, 1–2 times per week, 3–4 times per week, 5–6 times per week, once every 15 days, once a month, or never. To standardize intake across different frequencies, the reported consumption amounts were multiplied by the following coefficients: 3 for each meal, 1 for each day, 0.125 for 1–2 times per week, 0.485 for 3–4 times per week, 0.7865 for 5–6 times per week, 0.066 for once every 15 days, and 0.033 for once per month. This allowed us to calculate the average daily consumption of each food. The corresponding AGE, CML, CEL, and MG-H1 values were then determined by multiplying the daily intake (g or mL) of each food by its AGE content in the database [17,18]. The dietary AGE intake of the participants was calculated using a database prepared by Uribarri et al. that contains the AGE content of 549 foods [17]. The daily intake of AGEs consumed by the participants was calculated using this database [17]. The dietary intakes of carboxymethyl lysine (CML), carboxyethyl lysine (CEL), and methylglyoxal-derived hydroimidazolone-1 (MG-H1) were calculated using the database developed by Scheijen et al. [18]. In this database, similar to the dietary AGE database, the CML, CEL, and MG-H1 values for each food have been calculated [18]. For foods not included in the database, calculations were made using foods that were included in the list that were similar in terms of content, preparation, and cooking methods.

### 2.5. Laboratory Investigations

Blood samples were collected in the early follicular phase (days 2–5 of menstruation) after 10–12 h of fasting, and they were centrifuged at 3000 rpm for 10 min. The serum samples were collected in Eppendorf tubes and stored at −80 °C until the analysis took place. The levels of AGEs (CML, soluble RAGE, and methylglyoxal), inflammation markers (TNF-α and hs-CRP), and hormones (total testosterone, SHBG, and insulin) in the serum samples were measured using an enzyme-linked immunosorbent assay (ELISA). We used the following kits from the Bioassay Technology Laboratory (BT-Lab; Shanghai, China): Human Carboxymethyl Lysine ELISA Kit (Standard Curve Range: 20–3000 ng/mL, Cat.No E1413Hu), Human Soluble Receptor for Advanced Glycation End Product ELISA Kit (Standard Curve Range: 0.05–20 ng/mL, Cat.No E0027Hu), Human Methylglyoxal ELISA Kit (Standard Curve Range: 0.5–200 ng/mL Cat.No E4106hu), Human Tumor Necrosis Factor-α ELISA Kit (Standard Curve Range: 3–900 ng/L, Cat.No E0082Hu), Human High-Sensitivity C-Reactive Protein ELISA Kit (Standard Curve Range: 0.1–40 ng/mL Cat.No E1805Hu), Human Insulin ELISA Kit, The Bioassay Technology Laboratory (Standard Curve Range: 0.2–60 mIU/L, Cat.No E0010Hu), Human Sex Hormone-Binding Globulin ELISA Kit (Standard Curve Range: 0.5–200 nmol/L Cat.No E1011Hu), and Human Testosterone ELISA Kit (Standard Curve Range: 0.5–150 nmoL/L Cat.No EA0032Hu). Fasting blood glucose measurements were taken at the Famagusta Medical Center Laboratory. The free androgen index (FAI) and homeostatic model assessment of insulin resistance (HOMA-IR) measurements were taken according to the following standardized equations: FAI = [Total testosterone (mmol/L)/SHBG (nmol/L) × 100] [19]. HOMA-IR = [fasting insulin (uIU/mL) × fasting plasma glucose (FPG) (mg/dL)/405] [20].

### 2.6. Statistical Analysis

Descriptive statistics were used to summarize all the study variables. Categorical variables were presented as frequencies and percentages, while continuous variables were reported as mean ± standard deviation and median (minimum–maximum). The normality of continuous variables was assessed using both the Kolmogorov–Smirnov and Shapiro–Wilk tests. Group comparisons were performed using the independent-samples *t*-test or the Mann–Whitney U test, based on the fulfillment of parametric test assumptions. To assess associations between continuous variables, correlation analyses were conducted and visualized using heatmaps. The diagnostic performance of the selected variables was evaluated using receiver operating characteristic (ROC) curve analysis. Optimal cut-off values were determined based on the Youden Index, and corresponding sensitivity and specificity values were reported. All statistical analyses and visualizations were conducted using R software (version 4.3.2; R Core Team, Vienna, Austria) via RStudio (version 2024.12.1+563, Posit Software, Boston, MA, USA). Statistical significance was set at *p* < 0.05.

## 3. Results

Table 1 shows the demographic data, anthropometric measurements, and dietary AGE intakes of the group comprising individuals with PCOS and the control group. The mean age of the women (23.49 ± 2.92 years) with polycystic ovary syndrome was similar to that of the control group (22.55 ± 2.39 years) (*p* > 0.05). The waist-to-hip ratio was found to be higher in the PCOS group (0.88 ± 0.10) than in the control group (0.83 ± 0.06) (*p* = 0.009). The PCOS group had a significantly higher daily dietary AGE intake (13,191.05 ± 3360.12 kU/day) compared with the control group (11,740.28 ± 2940.61 kU/day) (*p* = 0.035). There was no significant difference between the groups in terms of the amounts of dietary CML, CEL, and MG-H1 (*p* > 0.05).

The serum CML levels were statistically significantly higher in the PCOS group (883.53 ± 582.29 ng/mL) than in the control group (651.19 ± 443.10 ng/mL) (*p* = 0.045). There was no significant difference between the groups regarding serum sRAGE levels (PCOS = 4.69 ± 3.19 ng/mL; control = 5.95 ± 3.93 ng/mL) (*p* = 0.063). However, the serum CML/sRAGE ratio was found to be statistically significantly higher in the PCOS group (413.94 ± 1114.79) compared with the control group (143.24 ± 124.71) (*p* = 0.002) (Table 2).

**Figure 1:**
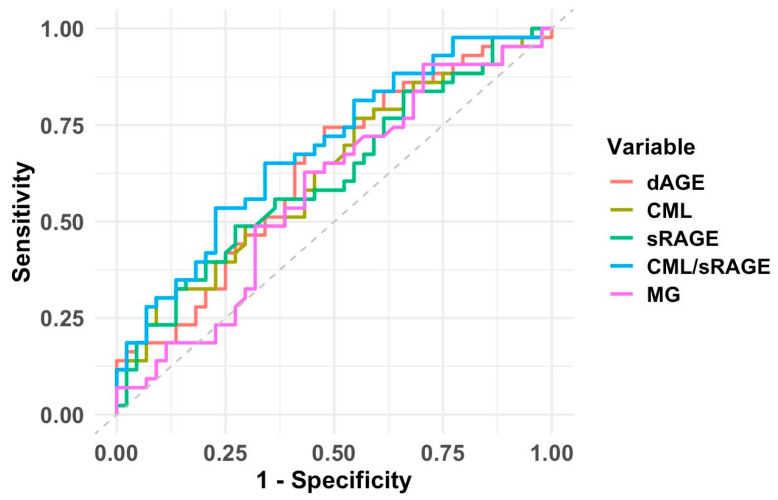
ROC curve of the markers evaluated for PCOS prediction. Abbreviations: dAGEs: dietary advanced glycation end products; CML: N-carboxymethyl lysine; sRAGE: soluble receptor for advanced glycation end products; MGO: methylglyoxal.

The findings from the ROC analysis showed that the CML/sRAGE ratio may be the strongest biomarker associated with the risk of developing PCOS (AUC = 0.688; cut-off point = 140.91 ng/mL). The sensitivity and specificity of this parameter were calculated to be 65.0% and 66.0%, respectively. The cut-off points for dietary AGE intake, serum CML, and sRAGE levels that may be associated with the risk of developing PCOS were found to be 11,359.06 kU/day (AUC = 0.636, sensitivity 74.0% and specificity 52.0%), 417.50 ng/mL (AUC = 0.625, sensitivity 77.0% and specificity 45.0%), and 3.11 ng/mL (AUC = 0.616, sensitivity 49.0%, and specificity 73.0%), respectively. The serum MGO cut-off point for an increased risk of PCOS development was 26.99 ng/mL (AUC = 0.616, sensitivity 91.0%, and specificity 30.0%). The results of the Youden Index for the parameters in the ROC analysis were 0.310 for the CML/sRAGE ratio, 0.260 for dietary AGE intake, 0.220 for serum CML, 0.220 for serum sRAGE, and 0.210 for serum MGO. The CML/sRAGE ratio has the highest Youden Index value, confirming that this biomarker is the best choice in terms of overall test performance (Figure 1).

**Figure 2:**
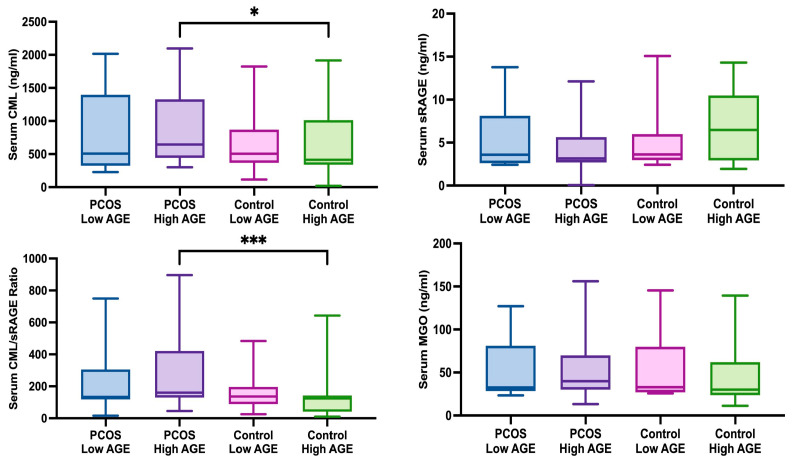
Comparison of serum AGE levels according to the dietary AGE intake cut-off value. Mann–Whitney U test * *p* < 0.05; *** *p* < 0.001. Abbreviations: dAGEs: dietary advanced glycation end products; CML: N-carboxymethyl lysine; sRAGE: soluble receptor for advanced glycation end products; MGO: methylglyoxal.

Based on the cut-off value for dietary AGE intake determined by the ROC analysis (11,359.06 kU/day), individuals in both the PCOS and control groups were stratified, and subgroup analyses were performed. According to the results of the subgroup analysis, the serum CML levels (919.58 ± 586.75 ng/mL) and CML/sRAGE ratios (483.29 ± 1284.74) were significantly higher in PCOS patients with high dietary AGE intake compared with those in the control group (634.87 ± 469.30 ng/mL and 124.23 ± 128.82, respectively) (*p* = 0.030 and <0.001, respectively) (Figure 2). The results of serum AGE comparisons based on dietary AGE intake cut-off are shown in Supplementary Table S1.

**Figure 3:**
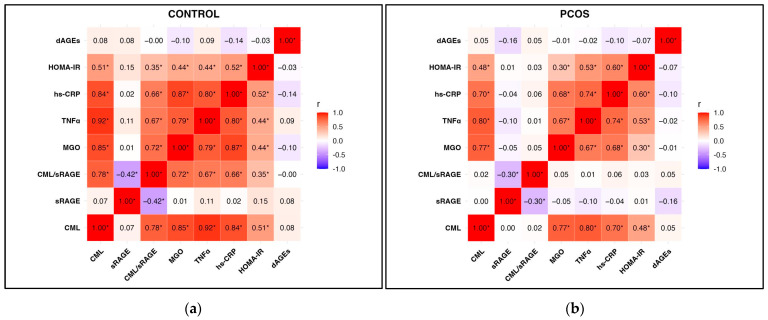
Pearson heatmap of metabolic and dietary parameters. Darker blue indicates a negative correlation, and red indicates a positive correlation; * *p* < 0.05. (**a**) Control group correlation analyses; (**b**) PCOS group correlation analyses. Abbreviations: dAGEs: dietary advanced glycation end products; CML: N-carboxymethyl lysine; sRAGE: soluble receptor for advanced glycation end products; MGO: methylglyoxal; hs-CRP: high-sensitivity C-reactive protein; TNF-α: tumor necrosis factor-alpha; HOMA-IR: homeostatic model assessment of insulin resistance.

Figure 3a shows the correlation analyses of the control group. The results showed a strong positive correlation between the serum CML levels and both the TNF-α and hsCRP levels (r = 0.92 and r = 0.84, respectively) (*p* < 0.001). The TNF-α and hsCRP levels were found to have a strong positive correlation with the serum MGO levels (r = 0.79 and r = 0.87, respectively) (Figure 3a, *p* < 0.001). On the other hand, there was no statistical correlation between dietary AGE intake and metabolic parameters in the control group.

Figure 3b shows the correlation analyses of the PCOS group. The TNF-α and hsCRP levels were found to have a strong positive correlation with the serum CML levels (r = 0.80 and r = 0.70, respectively) (*p* < 0.001). There was a moderately significant positive correlation between the serum MGO levels and the TNF-α and hsCRP levels (r = 0.67 and r = 0.68, respectively, *p* < 0.001).

We evaluated the relationship between hormone levels and serum AGEs in women with PCOS. We found a moderate positive correlation between serum SHBG and serum sRAGE levels (r = 0.504; *p* = 0.01) and a moderate positive correlation between serum testosterone and serum CML levels (r = 0.401; *p* = 0.008). There was a weak negative correlation between FAI levels and serum sRAGE levels (r = −0.346; *p* = 0.023).

## 4. Discussion

This study investigated the relationship between dietary AGE intake, serum AGE levels, and metabolic parameters in women with PCOS and a control group comprising women without PCOS. We found that dietary AGE intake, serum CML level, and CML/SRAGE ratio were higher in the women with PCOS compared with the control group. We determined the cut-off points for dietary AGE intake, serum CML, sRAGE, MGO, and the CML/sRAGE ratio for disease risk. It has been shown previously that these findings can be used in clinical risk assessments.

The dietary AGE intake of women with PCOS was found to be higher than that of the control group. Previous researchers have shown that a diet high in AGEs may lead to impaired ovarian function by affecting folliculogenesis and steroidogenesis in women [2,21]. The intake of dietary AGEs may play a role in the etiology and pathogenesis of PCOS by affecting serum AGE levels [5,10,22]. A review of human, animal, and cell studies found that dietary AGE intake may negatively affect the morphology or functioning of ovarian tissue, contributing to PCOS pathology [11]. In this study, the fact that the age and BMI levels of women in the PCOS and control groups were similar supports the hypothesis that AGE intake may contribute to the development of PCOS, independent of age and BMI.

AGEs are present in food and are also formed during the processing of food [7]. The AGE content of animal-derived foods that are cooked at a high temperature or in dry heat for a long time is particularly high [8]. A review of the literature reveals heterogeneity in the dietary assessment methods and databases used to estimate dietary AGE intake. Consequently, this variability makes it difficult to establish disease-specific recommendations for daily dietary AGE consumption [22,23]. For instance, when evaluating studies involving various disease groups, daily dietary AGE intake was found to range from 4000 to 24,000 kU/day, whereas in individuals without PCOS, this value was approximately 9000–23,000 kU/day [22]. In this study, the optimal dietary AGE intake cut-off point for PCOS risk was found to be 11,359.06 (kU/day), and 74.4% of women with PCOS and 47.7% of women without PCOS had dietary AGE intakes above this cut-off point. Intervention trials that restricted dietary AGE intake in women with PCOS have demonstrated reductions in PCOS-related metabolic complications. To reduce dietary AGE intake, it is very important for women with PCOS to use cooking methods, such as cooking in the food’s own juices, steaming, boiling, or cooking in a small amount of water [24,25].

The serum CML level is considered to be the best indicator of AGE level in the body [6,7]. In this study, the serum CML levels in women with PCOS were found to be higher than those in the control group. The elevated dietary AGE intake in women with PCOS may have contributed to their higher serum CML concentrations compared with the subjects without PCOS. In the meta-analysis by Bahreiny et al. [9], the serum AGE levels were found to be significantly higher in women with PCOS compared with the controls without PCOS. Diamanti-Kandarakis et al. [26] found that serum AGE levels were higher in women with PCOS who had a normal body weight and no insulin resistance compared with women without PCOS. The findings of both Bahreiny et al. [9] and Diamanti-Kandarakis et al. [26] are consistent with those of the present study. Mouanness et al. [27] stated that the use of serum AGE levels as a potential biomarker in the monitoring and treatment of metabolic complications in women with PCOS is of great importance. In this study, the optimal serum CML level for PCOS risk was determined to be 417.50 ng/mL, and 76.7% of the women with PCOS and 54.4% of the women without PCOS had serum CML levels above the value determined for PCOS risk.

Methylglyoxal is one of the most common precursors of AGE synthesis due to its high reactivity [28]. Due to the potential role that it plays in diabetes, cancer, and neurodegenerative diseases, MGO has been frequently investigated in recent years [29]. We could find only one study that examined the levels of MGO in women with PCOS [30], which found that the level of MGO was higher in individuals with PCOS compared with the control group. In the study by Song et al. [30], it was observed that the age, BMI, and HOMA-IR levels of the individuals were different between the PCOS and control groups. In this study, the similarity of the demographic characteristics and HOMA levels may have led to comparable serum MGO levels being observed between the PCOS and control groups. MGO levels increase only under conditions of marked hyperglycemia or advanced metabolic dysfunction. MGO does not show significant changes in diabetic individuals with mild to moderate metabolic disorders [31,32,33]. The serum MGO cut-off point, indicating an increased risk of developing PCOS, was found to be 26.99 ng/mL. The serum MGO levels were above the cut-off point for PCOS risk in 90.7% of the women with PCOS and 70.5% of the women without PCOS.

The soluble receptor for advanced glycation end products (sRAGE) circulates in the bloodstream and mitigates the harmful effects of AGEs. sRAGE binds to AGEs, preventing them from interacting with the AGE receptor (RAGE) and reducing the risk of cellular oxidative damage [34,35,36]. sRAGE can be found in blood and body fluids, including follicular fluid [6,37]. Studies have demonstrated that sRAGE levels in the follicular fluid of women with PCOS are significantly lower than in women without PCOS [9,37,38,39]. This finding suggests that sRAGE may support a reduction in follicular inflammation in PCOS by decreasing inflammatory markers in the follicular fluid [23]. In another study conducted on women with PCOS, individuals with lower sRAGE levels were found to have higher HOMA-IR and FAI values [40]. In a four-year prospective cohort study of women with PCOS, those who developed impaired glycemic control and metabolic syndrome had lower serum sRAGE levels and higher HOMA-IR levels [23]. However, after adjusting for BMI, FAI, and HOMA-IR, no differences were found in serum sRAGE levels [23]. In this study, no significant difference was found in the serum sRAGE levels between the PCOS group and the non-PCOS control group. The similarity of key clinical parameters that can influence serum sRAGE levels, such as BMI and HOMA-IR, between women with PCOS and the control group may have contributed to this result. In this study, having a serum sRAGE level above 3.11 ng/mL was identified as a potential risk factor for PCOS. The serum sRAGE levels exceeded this threshold in 65.1% of the women with PCOS and 61.4% of the women without PCOS.

The increase in serum AGE levels not only enhances oxidative stress but also contributes to the activation of the AGE–sRAGE interaction [41]. In this study, no differences were observed between the PCOS and control groups regarding inflammatory parameters such as TNF-α and hs-CRP. However, a strong correlation was identified between serum AGE levels and inflammatory parameters in both groups. Evidence from human, animal, and in vitro studies has shown that dietary AGEs may contribute to the development and progression of PCOS by activating the key intracellular signaling pathways that promote oxidative stress and the production of proinflammatory cytokines [10,41,42]. In rats fed a high-AGE diet for 12 weeks, increased inflammation was shown to impair ovarian function [21]. Gonzalez et al. [43] reported that both serum AGE levels and oxidative stress were elevated in women with PCOS who had normal blood glucose levels. Diamanti-Kandarakis et al. [44] suggested that the chronic inflammatory state in PCOS accelerates the accumulation of AGEs and that excessive AGE levels further exacerbate oxidative stress in tissues and organs. Elevated serum AGE levels can activate intracellular signaling via both receptor-dependent and receptor-independent mechanisms, leading to the increased production of reactive oxygen species and inflammatory cytokines [7]. For instance, the activation of NADPH oxidase, as well as the increased secretion of interleukins (IL-1, IL-6, IL-7, IL-8) and vascular cell adhesion molecule-1 (VCAM-1), has been shown to play a significant role in the initiation and progression of cellular inflammation [4,12,27].

It has been suggested that the serum levels of AGE, RAGE, and sRAGE may vary at different stages of diseases, and that neither elevated serum AGE levels nor decreased sRAGE levels alone can be considered reliable indicators of disease risk. Instead, the AGE/sRAGE or CML/sRAGE ratio has been identified as potentially critical for disease risk assessment [45,46,47,48,49]. In the present study, the CML/sRAGE ratio was found to have a greater discriminatory potential for possible PCOS risk compared with the other biomarkers examined (CML, sRAGE, and MGO). The optimal serum CML/sRAGE ratio for predicting PCOS risk was determined to be 140.91 ng/mL, with 62.8% of women with PCOS and 34.1% of women without PCOS having serum CML/sRAGE ratios above this threshold.

Compared with the controls without PCOS, individuals with PCOS exhibit decreased SHBG levels and increased free androgen levels [50]. The imbalance between these two hormones contributes to the severity of PCOS complications [51]. In the present study, a strong positive correlation was observed between serum sRAGE and serum SHBG levels, as well as between serum testosterone and serum CML levels. Consistent with our findings, previous studies have also reported a positive correlation between serum sRAGE and SHBG levels [23]. An increase in sRAGE levels and a decrease in CML levels in individuals with PCOS may lead to reduced oxidative stress and inflammation, thereby promoting higher SHBG levels and lower androgen hormones [40,50]. Various studies have shown that feeding rats a diet high in AGEs impairs folliculogenesis and steroidogenesis, thereby damaging ovarian function [21,27]. It has been determined that exposing human granulosa cells to AGEs may lead to RAGE-mediated anovulation associated with PCOS [52,53]. Therefore, monitoring CML and sRAGE levels in individuals with PCOS is of great importance.

Serum AGEs may play a role in the development of insulin resistance, a characteristic feature of PCOS, and actively contribute to PCOS pathogenesis [9]. In this study, a moderately strong, positive, and statistically significant association was also found between HOMA levels and both serum CML and MGO levels in both the PCOS group and the non-PCOS control group. Similarly, a meta-analysis reported a positive correlation between serum AGE levels and HOMA-IR [9]. A cell study conducted on human granulosa cells demonstrated that AGEs affect intracellular insulin signaling pathways and negatively impact glucose transport into the cell [54]. In animal studies, reducing dietary AGE intake has been shown to lower serum AGE levels and improve insulin sensitivity [55,56]. This result may be explained by the ability of AGEs to inhibit insulin clearance or enhance insulin secretion, thereby contributing to the development of mechanisms underlying insulin resistance [13].

This study has several limitations. First, as this study is based on observational data, the results cannot be directly interpreted in terms of causality. Therefore, further investigation of these findings in future longitudinal studies is warranted. Although the sample size was determined by power analysis and the groups were carefully matched for age and BMI, the relatively small sample size affects the generalizability of the results. Larger multicenter studies with more diverse populations are needed to improve the generalizability of our findings. Although a detailed and well-structured FFQ was used to estimate dietary AGE intake, dietary data were based on self-reported information, which may be subject to recall bias and misreporting. Another limitation of our study is that urinary AGE concentrations were not evaluated. For a more comprehensive and objective assessment of dietary AGE intake, future studies should consider measuring both plasma and urinary AGE levels. This combined approach would provide a more accurate and holistic evaluation of AGE exposure. Additionally, larger studies involving individuals from different ethnic backgrounds and various PCOS phenotypes are needed before dietary AGE and serum AGE biomarkers can be recommended for clinical applications. Finally, while the diagnosis of PCOS was made according to the Rotterdam criteria, this study did not classify participants based on their specific PCOS phenotypes.

One of the strengths of this study is that the women with PCOS and the control group were matched in terms of the participants’ age and BMI, which facilitated the interpretation of the biochemical parameters. In addition to serum AGEs, a wide range of biochemical parameters, such as insulin resistance, inflammation, and hormonal markers, were also evaluated. This allowed for a more detailed investigation of the relationships between these parameters. To our knowledge, apart from this study, there is only one other that has evaluated serum MGO levels in patients with PCOS, making this the second study to do so in this population; thus, we have made a unique contribution to the literature. In previous studies, it has been emphasized that evaluating serum CML or sRAGE levels alone is not sufficient in different disease groups. Finally, the assessment of the CML/sRAGE ratio, which is of critical importance for disease risk in women with PCOS, is considered another strength of this study.

## 5. Conclusions

In the management of patients with PCOS, the primary goals are to alleviate symptoms, prevent the development of potential complications, and slow down the progression of long-term complications. Reducing the dietary intake of advanced glycation end products (AGEs) is of great importance for both minimizing and preventing complications in women with PCOS and for reducing the risk of PCOS development and inflammation in women at risk for PCOS. Therefore, reducing dietary AGE intake is of critical importance for patients with PCOS. Avoiding high-temperature, dry-heat cooking methods, such as frying, roasting, baking, and grilling, and instead opting for low-temperature, moist-heat methods like boiling and steaming will help decrease dietary AGE intake. Additionally, regular monitoring of serum AGE levels in both women with PCOS and those at risk may help reduce potential health risks.

## Figures and Tables

**Table 1:** Comparison of demographic and anthropometric measurements and AGE intakes.

Variable	Control (*n* = 44)	PCOS (*n* = 43)	*p*
Age (years) •	22.00 (19.00–29.00)	23.00 (19.00–31.00)	0.134
BMI (kg/m^2^) •	26.40 (21.00–39.60)	26.40 (19.30–42.90)	0.750
Waist-to-hip ratio †	0.83 ± 0.06	0.88 ± 0.10	0.009 *
Dietary AGEs (kU/day) †	11,740.28 ± 2940.61	13,191.05 ± 3360.12	0.035 *
Dietary CML mg/day •	2.64 (1.31–9.66)	2.63 (1.26–5.81)	0.375
Dietary CEL mg/day •	2.74 (1.15–5.96)	2.20 (1.24–6.05)	0.366
Dietary MG-H1 mg/day •	21.91 (10.22–45.31)	20.12 (10.54–46.09)	0.647

† Data are normally distributed. Mean ± SD; *p*-value was calculated using Student’s *t*-test. • Data are not normally distributed. Median (min–max); *p*-value was calculated using the Mann–Whitney U test. * *p* < 0.05. Abbreviations: BMI: body mass index; AGEs: advanced glycation end products; CML: N-carboxymethyl lysine; CEL: N-carboxyethyl lysine; MG-H1: methylglyoxal hydroimidazolone.

**Table 2:** Biochemical characteristics.

Variable	Control (*n* = 44)	PCOS (*n* = 43)	*p*
Serum CML (ng/mL) **•**	457.80 (20.00–1916.50)	558.50 (227.10–2096.50)	0.045 *
Serum sRAGE (ng/mL) **•**	3.70 (1.93–15.07)	3.21 (0.05–13.78)	0.063
Serum CML/sRAGE ratio **•**	129.79 (10.36–643.12)	155.81 (16.48–7422.00)	0.002 *
Serum MGO (ng/mL) **•**	31.66 (11.18–145.46)	39.46 (13.29–156.02)	0.200
hs-CRP (ng/mL) **•**	5.62 (0.50–27.23)	6.34 (1.72–36.73)	0.368
TNF-α (ng/L) **•**	126.80 (55.10–660.20)	152.80 (71.80–702.10)	0.086
Fasting insulin (mIU/L) **•**	9.46 (4.01–22.72)	9.81 (6.41–48.28)	0.083
HOMA-IR **•**	1.82 (0.79–4.71)	1.86 (1.26–8.58)	0.145
SHBG (nmol/L) **•**	80.55 (32.20–118.10)	32.67 (18.76–81.86)	<0.001 *
Total testosterone (nmoL/L) **•**	28.18 (13.79–32.74)	59.42 (38.51–89.26)	<0.001 *
FAI **•**	1.28 (0.69–3.20)	5.56 (2.14–15.12)	<0.001 *

Data are normally distributed. Mean ± SD; *p*-value was calculated using Student’s *t*-test. • Data are not normally distributed. Median (min–max); *p*-value was calculated using the Mann–Whitney U test. * *p* < 0.05. Abbreviations: CML: N-carboxymethyl lysine; sRAGE: soluble receptor for advanced glycation end products; MGO: methylglyoxal; hs-CRP: high-sensitivity C-reactive protein; TNF-α: tumor necrosis factor-alpha; HOMA-IR: homeostatic model assessment of insulin resistance; SHBG: sex hormone-binding globulin; FAI: free androgen index.

## Data Availability

The original contributions presented in this study are included in the article. Further inquiries can be directed to the corresponding author.

## Supplementary Materials

The following supporting information can be downloaded at: https://www.mdpi.com/article/10.3390/jcm14165803/s1, Table S1: Comparison of serum AGE levels according to the dietary AGE intake cut-off value.

## Abbreviations

PCOS	Polycystic ovary syndrome
AGEs	Advanced glycation end products
dAGEs	Dietary advanced glycation end products
CML	Carboxymethyl lysine
CEL	Carboxyethyl lysine
MG-H1	Methylglyoxal-derived hydroimidazolone-1
ELISA	Enzyme-linked immunosorbent assay
sRAGE	Soluble receptor for advanced glycation end products
MGO	Methylglyoxal
SHBG	Sex hormone-binding globulin
FAI	Free androgen index
HOMA-IR	Homeostatic Model Assessment of Insulin Resistance
BMI	Body mass index
TNF-α	Tumor necrosis factor-α
hs-CRP	High-sensitivity C-reactive protein
ROC	Receiver operating characteristic
AUC	Area under the curve
kU	kilo-Unit
