# Supplementary material for: Are Dietary and Serum Advanced Glycation End Products (AGEs) Potential Contributors to Inflammation in Women with Polycystic Ovary Syndrome?
Source: J Clin Med. 2025 Aug 16;14(16):5803. doi: 10.3390/jcm14165803 (PMC12387888; doi:10.3390/jcm14165803)
Supplement: Supplementary file 1 [file jcm-14-05803-s001.zip › jcm-3787189-supplementary.pdf]

Supplementary Table S1. Comparison of serum AGE levels according to the dietary AGE intake cut-off value

Variables	PCOS (n=43)			Control (n=44)			p^a	p^b
	**Low AGE (n=11)**	**High AGE (n=32)**	**p**	**Low AGE (n=23)**	**High AGE (n=21)**	**p**		
Serum CML (ng/ml)•	507.80 (715.35)	644.15 (869.83)	0,428	504.50 (446.35)	412.50 (601.30)	0,545	0,754	**0,030** *
Serum sRAGE (ng/ml) •	3.59 (4.10)	3.16 (2.44)	0,824	3.63 (2.49)	6.46 (7.42)	0,549	0,537	0,066
Serum CML/ sRAGE ratio•	133.43 (111.92)	160.35 (274.96)	0,276	136.41 (100.72)	123.39 (97.99)	0,198	0,612	**<0,001***
Serum MGO (ng/ml)•	32.52 (44.48)	39.99 (35.36)	0,587	32.85 (48.58)	30.02 (15.39)	0,213	0,797	0,084

• Data are not normally distributed. Median (IQR); p value was calculated using Mann Whitney U test. *p<0.05

p^a: Comparison between groups within the Low AGE category as determined by the ROC curve cut-off value

p^b: Comparison between groups within the High AGE category as determined by the ROC curve cut-off value

Abbreviations: CML: N-carboxymethyl lysine; sRAGE: soluble receptor for advanced glycation end products; MGO: methylglyoxal;
